# Increased risk of Eustachian tube disorders in patients with sleep-disordered breathing: Erratum

**DOI:** 10.1097/MD.0000000000008002

**Published:** 2017-09-01

In the article, “Increased risk of Eustachian tube disorders in patients with sleep-disordered breathing”,^[1]^ which appeared in Volume 96, Issue 31 of *Medicine*, the affiliations should have appeared as follows:

^a^Department of Otolaryngology-Head and Neck Surgery, China Medical University Hospital, Taichung, Taiwan, ^b^Department of Sports Medicine, ^c^Management Office for Health Data, ^d^Department of Public Health, ^e^Institute of Biostatistics, ^f^Graduate Institute of Clinical Medical Science, China Medical University, Taichung, Taiwan, ^g^Department of Audiology and Speech-Language Pathology, Asia University, Taichung, Taiwan.

^∗^Correspondence: Yung-An Tsou, Graduate Institute of Clinical Medical Science, China Medical University, Departments of Otorhinolaryngology, China Medical University and Hospital, Audiology and Speech-Language Pathology, Asia University, No. 91, Hsueh-Shih Road, Taichung City 40402, Taiwan (R.O.C) (e-mail: d22052121@gmail.com).
